# Reversibility of Retinal Microvascular Changes in Severe Falciparum Malaria

**DOI:** 10.4269/ajtmh.14-0116

**Published:** 2014-09-03

**Authors:** Richard J. Maude, Hugh W. F. Kingston, Sonia Joshi, Sanjib Mohanty, Saroj K. Mishra, Nicholas J. White, Arjen M. Dondorp

**Affiliations:** Mahidol-Oxford Tropical Medicine Research Unit, Faculty of Tropical Medicine, Mahidol University, Rajthevee, Bangkok, Thailand; Centre for Tropical Medicine, Nuffield Department of Medicine, University of Oxford, Oxford, United Kingdom; College of Medicine and Veterinary Medicine, University of Edinburgh, Edinburgh, United Kingdom; Global Health Division, Menzies School of Health Research and Charles Darwin University, Casuarina, Northern Territory, Australia; Ispat General Hospital, Rourkela, Orissa, India

## Abstract

Malarial retinopathy allows detailed study of central nervous system vascular pathology in living patients with severe malaria. An adult with cerebral malaria is described who had prominent retinal whitening with corresponding retinal microvascular obstruction, vessel dilatation, increased vascular tortuosity, and blood retinal barrier leakage with decreased visual acuity, all of which resolved on recovery. Additional study of these features and their potential role in elucidating the pathogenesis of cerebral malaria is warranted.

The pathogenesis of coma in falciparum malaria and its rapid reversibility are potential targets for adjunctive therapies, but they are not well-understood. Microvascular obstruction is probably an important contributor. The brain microvasculature is relatively inaccessible; it can be studied in detail only at post-mortem. Similarly, microvascular obstruction in the retina is thought to be a major contributor to the unique retinopathy of severe falciparum malaria, and, because it is easily visualized in living subjects, in-depth study is providing new and valuable insights. We describe an adult patient with cerebral malaria who had prominent retinal changes with some previously unrecognized features that resolved on recovery.

A 24-year-old male truck driver from Orissa, India was admitted with severe *Plasmodium falciparum* malaria (parasitemia = 0.3%) with coma, generalized convulsions, hyperlactatemia, renal failure, and black urine. He had no prior medical history. Retinal photography showed bilateral patchy macular whitening with corresponding capillary non-perfusion and leakage of fluorescein caused by blood retinal barrier breakdown on fluorescein angiography (Figure 1). He was treated with intravenous artesunate, and from recovery of consciousness on day 3 to discharge, his visual acuity was markedly reduced (counting digits only), with loss of red–green color vision. Repeat examination on day 55 showed that the retinal changes, angiogram abnormalities, and visual deficits had resolved (acuity 6/9 bilaterally and normal color vision). Blood vessel tortuosity measured in three arteries and three veins by a single blinded observer tracing the center line of vessels between branch points in matched pairs of retinal photographs using Adobe Photoshop CS4 (Adobe Systems, San Jose, CA)^1^ was greater on day 0 than day 55 (mean ratios of vessel widths measured at 10 points in each vessel; 1.226 in arteries and 1.172 in veins; vessel lengths were 1.043 and 1.035). These differences are similar to those found previously in diabetic macular edema.^1^

**Figure 1.**
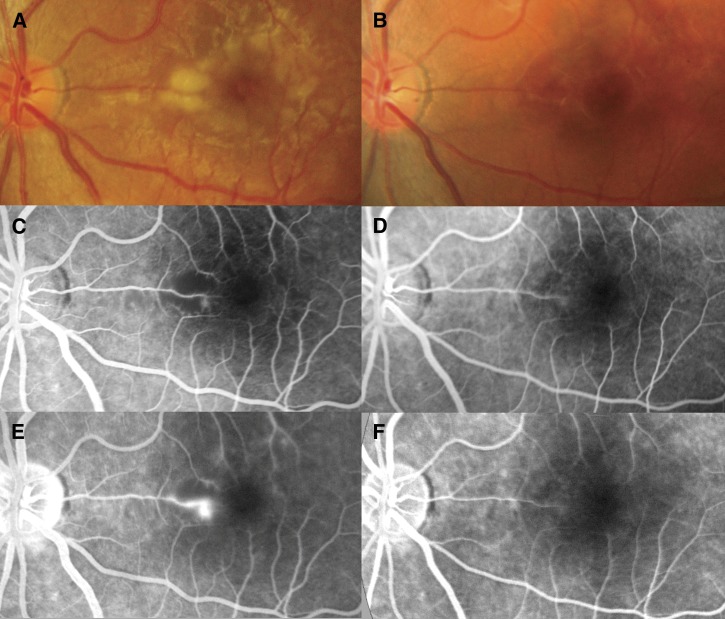
(**A** and **B**) Retinal photographs and (**C** and **D**, arterial phase; **E** and **F**, late phase) fluorescein angiograms of the left eye. On day 3, increased vessel thickness and tortuosity plus (**A**) patchy macular whitening with corresponding areas of (**C**) reduced perfusion and (**E**) fluorescein leakage were seen. On day 55, normal vessels, (**B**) no whitening, and (**D**) normal perfusion around the fovea with (**F**) no leakage of fluorescein were seen.

Malarial retinal whitening is thought to be caused by hypoxic opacification of the retina after obstruction of small blood vessels by sequestered parasites.^2^,^3^ It is similar to patchy ischemic retinal whitening (PIRW), a transient early sign of central retinal vein occlusion (CRVO)^2^ thought to represent intracellular edema of overlying retinal intermediary neurones.^4^ The degree of retinal whitening in adults and children correlates with severity of malaria and peripheral blood lactate.^5^,^6^ Hyperlactatemia is common in severe malaria and at least partly caused by obstruction of the systemic microcirculation by sequestered parasites. Cerebrospinal fluid lactate concentrations are also raised in cerebral malaria, and in those cases it is predictive of mortality.^7^

The appearance and distribution of retinal whitening are unique to severe falciparum malaria. Typically, there are multiple small lesions most prominent in the macula, particularly temporal to the fovea. This area is a watershed between the superior and inferior retinal vascular arcades and particularly vulnerable to ischemic insults. Midperipheral involvement in malaria distinguishes it from PIRW, Purtscher's retinopathy, and cotton wool spots (sometimes also seen in malaria), which are distributed particularly around the optic disk and typically more opaque. Malarial retinopathy is considered reversible,^8^,^9^ but this case is the first published photographic evidence of reversibility.

The angiogram in this patient showed that the whitening corresponds closely to capillary non-perfusion. This finding has not been described previously in adults but is common in Malawian children with cerebral malaria.^10^ Post-mortem studies in Malawi have found retinal blood vessels in cerebral malaria to be packed with sequestered parasites,^11^ similar to findings in the brain in adults.^12^ Because retinal whitening^9^ and central nervous system (CNS) sequestration^13^,^14^ are particularly prominent in patients with malarial coma (cerebral malaria), this finding suggests that small blood vessel CNS ischemia plays a major role in pathogenesis. In survivors, malarial coma is rapidly reversible and, as seen in the retina in this case, reversal of blood vessel obstruction is a plausible contributor.

This patient had mildly increased tortuosity of retinal blood vessels that decreased on recovery. Although increased vascular tortuosity has not been well-described in malaria, it is a recognized feature of other vascular occlusive diseases of the retina. Vessel tortuosity is caused by a combination of vessel dilation from radial stretching and the vessel taking a more serpentine path because of longitudinal stretching.^15^ Several pathogenic mechanisms have been proposed for increased retinal vascular tortuosity. They include (1) increased blood flow in anemia, (2) early angiogenesis caused by ischemia or inflammation and (3) dysregulation of vascular tone caused by microvascular obstruction and relative hypoxia in diabetic retinopathy, and (4) venous congestion causing elevated vascular pressure and dilatation of blocked vessels in CRVO and raised intracranial pressure resulting in central retinal vein compression. In malaria, anemia is common, uninfected red blood cells have reduced deformability, and sequestered parasites cause microvascular and venular obstruction. Angiogenesis is probably unimportant over the short timescale.^1^

Increased vascular tortuosity has not been well-described previously in severe falciparum malaria, possibly because the normal appearance of retinal vessels varies significantly between individuals and subtle changes are difficult to identify. Ophthalmoscopy revealed engorgement and tortuosity of retinal veins in 26% of children with cerebral malaria in Ghana, which mostly resolved by 1 week.^8^ In our patient, comparison of retinal photographs provided a more objective measure. Means of quantifying vessel tortuosity using computer-aided image processing are under development.

The angiogram in this patient showed focal leakage of fluorescein across the blood–retinal barrier (BRB) in areas of non-perfusion, suggesting a common etiology. The BRB is analogous to the blood–brain barrier, which is also mildly disrupted in cerebral malaria. Leakage from larger retinal vessels crossing ischemic areas is a well-known phenomenon in retinal ischemia. The significance of this finding as a contributor to the pathogenesis of malarial coma is not known. More angiographic studies are needed.

This patient had decreased visual acuity, which had resolved at follow-up. Although it is not possible to give a cause, it is the first report of an association between macular retinal whitening and decreased visual acuity with subsequent recovery.

Additional studies of malarial retinopathy have great potential to enhance our understanding of vascular changes in severe malaria. To maximize their impact, studies should use retinal photography, where possible, to allow detailed examination of the full range of fundus signs by multiple blinded observers. This examination should be done both acutely and at follow-up. Fluorescein angiography provides a highly detailed map of CNS retinal perfusion. There is a need for additional detailed studies to include assessment of vascular tortuosity to investigate its role as a potential early and sensitive marker in studies of severe malaria.

The rate of reversibility of malarial retinopathy has potential as an end point in intervention studies of severe malaria, particularly for adjunctive therapies that directly target the pathogenesis. Additional information on the speed of reversibility of the various components of malarial retinopathy is needed, and studies are underway to investigate this.
